# Engineering of MSCs sheet for the prevention of myocardial ischemia and for left ventricle remodeling

**DOI:** 10.1186/s13287-023-03322-7

**Published:** 2023-04-25

**Authors:** Dehua Chang, Xiaotong Yang, Siyang Fan, Taibing Fan, Mingkui Zhang, Minoru Ono

**Affiliations:** 1 Department of Cell Therapy in Regenerative Medicine, The University of Tokyo Hospital, 7-3-1 Hongo, Bunkyo-ku, Tokyo, 113-8655 Japan; 2 BOE Regenerative Medicine Technology Co., Ltd., No. 9 JiuXianQiao North Road, Beijing, 100015 China; 3 Heart Center and Beijing Key Laboratory of Hypertension, Beijing Chaoyang Hospital, Capital Medical University, Beijing, 100020 China; 4 Children Heart Center, Fuwai Central China Cardiovascular Hospital, No. 1 Fuwai Road, Zhengzhou, 450018 China; 5 Heart Center, First Hospital of Tsinghua University, No. 6 JiuXianQiao 1st Road, Beijing, 10016 China; 6 Department of Cardiac Surgery, The University of Tokyo Hospital, 7-3-1 Hongo, Bunkyo-ku, Tokyo, 113-8655 Japan

**Keywords:** Tissue engineering, Mesenchymal stem cell, Cell sheet, Heart failure, Regenerative medicine, Cardiac regeneration, Remodeling

## Abstract

Tissue engineering combines cell biology and material science to construct tissues or organs for disease modeling, drug testing, and regenerative medicine. The cell sheet is a newly developed tissue engineering technology that has brought about scaffold-free tissue and shows great application potential. In this review, we summarized recent progress and future possibilities in preclinical research into and clinical applications of cell sheets fabricated by differing cell types from various sources for cardiac tissue repair, and the manufacturing strategies and promising application potential of 3D cell-dense tissue constructed from cell sheets. Special attention was paid to the mechanisms of mesenchymal stem cell (MSC) sheets in the prevention of myocardial ischemia and left ventricle remodeling. Comparing MSCs sheets with other types of cell sheets and 3D cardiac tissues, engineering tissues' potential safety and effectiveness concerns were also discussed.

## Introduction

Heart failure (HF) remains a leading cause of morbidity and mortality worldwide [1–3]. It is estimated that there are nearly 64 million patients with HF globally, and the number increases every year [4, 5]. Ischemic heart disease is the principal etiology of HF. Myocardial ischemia (MI) occurs when blood flow to the heart is reduced, preventing the heart muscle from receiving enough oxygen. MI can directly cause myocardial cell apoptosis, which results in an inflammatory response and fibrosis formation. After the acute phase of MI, reduced heart function leads to a progressive change in the shape and size of the left ventricle (LV), worsening the prognosis of patients with HF. Cardiac remodeling and fibrosis occur, and the myocardium is eventually replaced by fibrotic scar tissue [6]. The effective procedure for end-stage HF is heart transplantation or an artificial heart. Heart transplantation is a limited supply of donors’ hearts and is also accompanied by associated cardiovascular risk factors. While transplantation of an artificial heart incurs risks of postoperative bleeding, infection, and other complications. Moreover, the high medical costs including charges for the surgical procedure, device, and continuing medical surveillance also bring a huge economic burden to the patients [7].

With the development of tissue engineering and regenerative medicine, the idea of heart tissue regeneration for HF treatment has emerged in the last twenty years. Bioactive cardiac patches, heart tissue scaffolds, and cell therapy came into being, accompanied by rapid progress in modern medical technologies, materials, and cytobiology [8]. Among them, cell therapy is in the spotlight for its “live drug” identity positioning, which can work in vivo by paracrine modulation, cell migration, and differentiation. Cell suspension injection is a common cell application route, due to its convenient delivery. But in general, fewer than 10‒20% of injected cells are available in the injured area within a few hours or days after delivery, and only a few cells actively engraft in the affected tissue, limiting their therapeutic efficacy [9]. One way to improve cell efficacy is combining cells with scaffolds such as collagen, which provides mechanical support for natural tissues, or with synthetic, biodegradable polymers. However, the foreign body reaction and the inflammation caused by scaffolds composed of natural or artificial materials are inevitable. Much remains to be done to perfect the mechanical properties of the scaffold.

A cell sheet technology developed by Okano’s team made it possible to construct a scaffold-free two-dimensional (2D) cell sheet. A thermo-responsive culture dish was used to proliferate cells and to harvest a monolayer cell sheet, by reducing culture temperature from 37 °C to less than 32 °C [10, 11]. Unlike traditional proteolytic enzymes involved in cell harvesting strategy, cell adhesion proteins, cell–cell junction proteins, and an extracellular matrix (ECM) can be retained through cell sheet technology, which can improve colonization rates and survival times after cell transplantation [12]. Several types of cells have been used to fabricate cell sheets. They were used for heart tissue repair and in the treatment of many other diseases, such as corneal surface reconstruction, esophageal regeneration, pulmonary air leakage repair, and periodontal regeneration (Table 1). For heart tissue repair, the most commonly used cell types include cardiomyocytes (CMs), autologous skeletal myoblasts (SMs), mesenchymal stem cells (MSCs), and induced pluripotent stem cell-derived cardiomyocytes (iPS-CMs) [13]. Skeletal muscle-derived myoblasts cell sheets were even used clinically to cure HF caused by dilated cardiomyopathy in both children and adults [14–16].

**Table 1:** Clinical trials of cell sheets from various sources for the repair of various tissues

Tissue	Applications	Cell source	Mono- or multi-layer	Organization	Reference
Heart	End-stage DCM patients with LVAS	Autologous myoblasts	4 sheet layers	Osaka University Graduate School of Medicine	[19]
	Severe heart failure (ICM and DCM)	Autologous myoblasts	3‒4 sheet layers	Osaka University Graduate School of Medicine	[15, 20, 21]
	Pediatric heart failure	Autologous myoblasts	1 sheet layer	Osaka University Graduate School of Medicine	[16]
	Ischemic cardiomyopathy	Allogeneic iPS-derived cardiomyocytes	Unreported	Osaka University Graduate School of Medicine	[22]
Eye	Unilateral corneal epithelial stem cell deficiency	Autologous corneal epithelial cells	Multi-cell layers	Keio University School of Medicine Graduate School of Medicine, The University of Tokyo	[23, 24]
	Limbal stem-cell deficiency	Autologous oral mucosal epithelial cells	Multi-cell layers	Osaka University Graduate School of Medicine Tohoku University Graduate School of Medicine Graduate School of Medicine, The University of Tokyo Ehime University Graduate School of Medicine	[25]
		iPS-derived corneal epithelial cells	Unreported	Osaka University Graduate School of Medicine	[26]
	Intractable keratoconjunctival disease	Autologous oral mucosal epithelial cells	Unreported	Kyoto Prefectural University of Medicine	[27]
	Exudative age-related macular degeneration	Autologous iPS-derived RPE cells	1 sheet layer	RIKEN	[28]
Esophageal	Preventing strictures after ESD	Autologous oral mucosal epithelial cells	4‒5 cell layers	Nagasaki University Hospital Tokyo Women’s Medical University	[29–31]
	Preventing anastomotic re-stricture after repair of congenital esophageal atresia	Autologous oral mucosal epithelial cells	Unreported	National Center for Child Health and Development	[32]
Cartilage	Cartilage regeneration	Autologous chondrocytes	3 sheet layers	Tokai University School of Medicine	[33]
		Allogeneic chondrocytes	3 sheet layers	Tokai University School of Medicine	[34]
Periodontal	Periodontal regeneration	Autologous periodontal ligament cells	3 sheet layers	Tokyo Women’s Medical University	[35]
		Allogeneic periodontal ligament-derived mesenchymal stromal cells	Unreported	Tokyo Medical and Dental University	[36]
Ear	Middle ear mucosal regeneration	Autologous nasal mucosal epithelial cells	Multi-cell layers	The Jikei University School of Medicine	[37, 38]
Lung	Sealing of lung air leaks	Autologous dermal fibroblasts	1‒3 cell layers	Tokyo Women’s Medical University	[39]

*LVAS* left ventricular assist system, *iPS* induced pluripotent stem cell, *RPE* retinal pigment epithelium, *ICM* ischemic cardiomyopathy, *DCM* dilated cardiomyopathy, *ESD* endoscopic submucosal dissection

Besides the 2D application of cell sheets, three-dimensional (3D) cell-dense tissue can be constructed by cell sheet stacking [17]. Blood vessels can then be sandwiched into slabs of 3D cardiac tissues, in order to better mimic heart tissue structure or to achieve more complex functions [18]. The 3D cell-dense cardiovascular tissues provide a possible solution for heart tissue repair or regeneration and can also be used in pharmacological modeling.

**Fig. 1:**
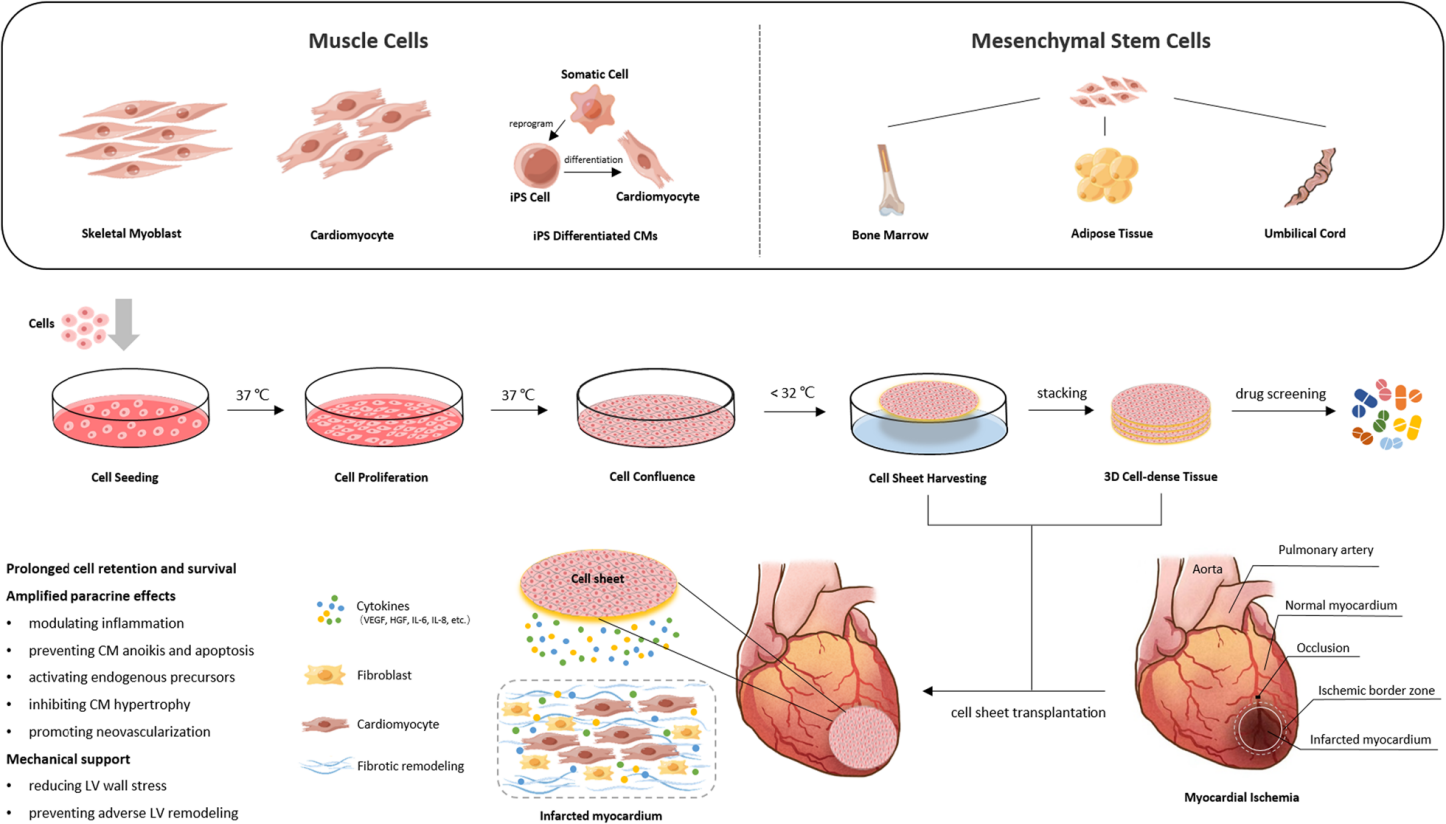
Schematic diagram of cell sheets fabrication by differing cell types from various sources and their applications for ischemic heart disease

In this review, we mainly summarized the preclinical research into, and clinical applications of cell sheets constructed by different cell types and from various sources for cardiac tissue repair (Fig. 1). The manufacturing strategies and promising application potentials of 3D cell-dense tissue constructed from cell sheets are also summarized.

## Cell types and sources for cell sheet fabrication

### Preclinical studies of cell sheets for cardiac tissue repair

Cell sheets constructed from CMs or MSCs have attracted the most attention in fundamental research, as the former is the major component of the functional myocardium, and the latter has the greatest potential to be developed as off-the-shelf products. At present, the applications of CM and MSC sheets are in preclinical studies.

#### Cardiomyocytes (CMs) sheet

Cardiomyocytes should be the ideal cell source for cardiac tissue construction, as they are the fundamental contractile units of the myocardium and occupy most of the myocardial volume. However, CMs are terminally differentiated, i.e., losing proliferative capacity soon after birth, and rarely dividing during adulthood. Therefore, in basic research, newborn rats’ CMs were usually used for cell sheet construction and cell sheet-based tissue engineering [40].

##### Construction of functional myocardial tissue with CM sheet

CMs isolated from neonatal rats were cultured to form monolayer cell sheets, and 3D cardiac tissues were then fabricated by stacking CM sheets layer by layer [41, 42]. The CM sheets contain electrically coupled cells, and when stacked, can form both electrical and morphological connections between layers [41, 43]. The resulting 3D cardiac tissue is a functional myocardial tissue with electro-mechanical communication between CMs, resembling the native myocardium. Due to the importance of vascularization in 3D-engineered tissues, sufficient pre-endothelialization of the CM sheets may be critical for transplantation and heart repair [40]. It was found that CM sheets with microvascular networks can be derived from CMs co-cultured with endothelial cells. After being transplanted into infarcted rats, these sheets exhibited higher livability than the transplanted CMs, and the endothelial cells contributed to vascular network formation in the host myocardium. When transplanted to the surface of the impaired rat’s heart, CM sheets showed histological and electrical integration with the recipient’s heart and thereby elicited synchronized beating between the transplanted cardiac tissue and the recipient’s heart [40, 42]. In a study by Okano’s team, four sheets were laid on top of each other until they fused and were then implanted under the subcutaneous tissues of immunodeficient rats. Six months later, the engineered cardiac tissue was beating, and blood vessels had permeated it. The heart tissue-like structures can be observed to pulse spontaneously in vitro [44].

##### Application of CM sheet in myocardial ischemia

The potential of CM sheets and related 3D cardiac tissues in the regeneration of impaired myocardium is now under investigation, mostly in animal experiments [40, 42]. Transplanted CM sheets were found to be able to bridge the barrier between the myocardial tissue graft and the recipient’s heart, with an improvement in host ejection fraction and inhibition of left ventricular dilatation, thus preventing cardiac dysfunction and HF. In a study by Sawa et al., CM sheets integrated with an impaired myocardium showed improved cardiac performance in a model of rats with ischemic myocardium [42]. CMs from neonatal rats were cultured on a temperature-responsive dish (Poly(N-isopropyl acrylamide)-grafted polystyrene) and were then harvested as square cell sheets. By stacking two such monolayer sheets, a tissue-engineered contractile CM sheet was obtained, which resembled homogeneous heart-like tissue in cross-section. After implantation of the CM sheets onto the infarcted myocardium, cardiac performance was significantly ameliorated, as demonstrated by echocardiography. Additionally, the threshold for the pacing of the recipient’s heart was lower than in fibroblast sheet implantation and in the control group.

#### Mesenchymal stem cells (MSCs) sheet

MSCs are adult stem cells that exist in many kinds of human tissues. Due to their accessibility and high proliferative potential, and their immunomodulatory, anti-inflammatory, and pro-angiogenic properties, MSCs have been widely investigated and are used in cell therapy and tissue engineering for cardiac regeneration [45, 46]. Moreover, the low immunogenicity enables allogeneic MSCs to be stored in cell banks for off-the-shelf applications. Many preclinical studies have shown that MSC sheets can significantly improve cardiac function [47]. Among the various MSC sources, bone marrow, adipose tissue, and the umbilical cord have attracted the most attention.

##### Bone marrow-derived mesenchymal stem cells (BM-MSCs) sheet

BM-MSCs are the most extensively investigated MSC type in donor MSC transplantation therapy for heart failure. Their therapeutic effects have been demonstrated in several studies, in which they were delivered via intracoronary injection or intramyocardial injection [48]. With the development of cell sheet technology, attempts were made to deliver BM-MSCs in sheet form, to reduce cell loss and improve cell viability. Narita et al. explored BM-MSC sheets therapy in a rat coronary artery ligation model. Compared with intramyocardial injection, the cell sheet technique clearly enhanced initial retention and following presence of BM-MSCs [49]. Subsequently, amplified paracrine effects, including increased neovascular formation, a decrease in fibrosis, attenuation of cardiomyocyte hypertrophy, and improvement of endogenous myocardial regeneration, were observed to recover the damaged heart and thereby improve therapeutic outcomes. Kawamura et al. evaluated the feasibility, safety, and effectiveness of human BM-MSC sheets in a porcine ischemic cardiomyopathy (ICM) model, in which remarkable improvement of cardiac function was demonstrated four weeks after transplantation of BM-MSC sheets, without mortality or complications [50]. The BM-MSC sheets preserved a large amount of ECM on the basal side, preventing anoikis and apoptosis from occurring due to cell detachment from the ECM. The paracrine effects of BM-MSCs, combined with the 2D structure of cell sheets, contributed to increased neovascularization in the infarct border area, attenuated LV remodeling, and improved cardiac functions thereby.

##### Adipose-derived mesenchymal stem cells (AD-MSCs) sheet

Adipose-derived mesenchymal stem cells (AD-MSCs) are easily obtained and expanded, and thus have emerged as a novel source of adult stem cells for the treatment of cardiovascular diseases. They have the potential to differentiate into cardiovascular lineages and can secrete a series of paracrine factors to promote neovascularization, reduce apoptosis, and inhibit fibrosis, which contributes to cardiac regeneration [51]. Miyahara et al. prepared monolayered AD-MSC sheets and transplanted them onto the scarred myocardium in a rat model with myocardial infarction [52]. AD-MSC sheets were readily engrafted to a scarred myocardium after transplantation and grew gradually in situ to become a thick stratum that included newly formed vessels, cardiomyocytes, and undifferentiated AD-MSCs. Moreover, the engrafted AD-MSC sheets reversed wall thinning in the scar area and thus improved cardiac function and survival. The effectiveness of AD-MSC sheets for heart repair was further verified in a study by Ishida et al. in a porcine chronic heart failure model [53]. After transplantation of triple-layered AD-MSC sheets for four weeks, a significant improvement in left ventricular ejection fraction (LVEF) was detected: from 41.4% ± 2.8% to 47.6% ± 2.9% in the sheet group, compared to from 38.9% ± 4.8% to 34.6% ± 1.9% in the control group. This was accompanied by collateral vessel growth into the ischemic area and a significant increase in capillary density in the peri-infarct ischemic myocardium. The treatment efficacy of AD-MSC sheets in myocardial ischemia animal models provided important foundations for future clinical studies.

##### Human umbilical cord-derived mesenchymal stem cells (UC-MSCs) sheet

Recently, the human umbilical cord has emerged as an attractive potential source of MSCs for use in regenerative medicine. UC-MSCs derived from a neonatal organ exhibited the greatest potential, as they not only possess features of all MSCs such as multi-lineage differentiation, paracrine functions, and immunomodulatory properties but also have other advantages, such as non-intrusive acquisition and low immunogenicity, making UC-MSCs suitable for allogeneic clinical application and even for the development of off-the-shelf products, with the help of cell bank establishment [54]. Similar to BM-MSCs and AD-MSCs, UC-MSCs were commonly delivered in past studies in suspension form via injection, and the development of cell sheet technology provided a new strategy for better efficacy. Guo et al. examined the therapeutic effects of UC-MSC sheets in a mouse MI model [55]. According to this research, UC-MSC sheets showed significantly prolonged local cell retention and survival, compared with cell suspensions, and can dramatically improve cardiac function by modulating post-MI inflammation, promoting angiogenesis, and reducing fibrosis [55]. When observed by an in vivo bio-luminescent imaging system, in ICR (Institute of Cancer Research) mice, human UC-MSC suspension remained for less than five days after transplantation, while the cell sheet retention time was more than nine days. This corresponded with the anti-HNA (human nuclei antigen) staining analysis result, in which human cells were found to have survived nine days after cell sheet transplantation. A decrease of Mcp1-positive monocytes and classical CD68-positive macrophages, and an increase of Cx3cr1-positive cells, was observed five days after cell sheet transplantation, which indicated that cell sheets can decrease inflammation-induced cardiac injury and prevent cardiac damage in the acute stage. Immunostaining of IB4 and α-SMA revealed that cell sheets promoted neovessel formation response in the border and infarct zones, and arteriole formation response mainly in the border zone. Additionally, Masson staining of heart samples harvested 28 days after MI showed that in the cell sheet transplantation group, the thickness of the LV wall was significantly improved in the border zone, with no significant difference in the infarct zone compared with the cell suspension and the MI-only groups. That means the cell sheet mainly promotes angiogenesis and decreases fibrosis in the MI border zone to improve cardiac function and alleviate pathogenic remodeling.

Moreover, according to recent study results obtained by the Beijing Oriental Electronics (BOE) regenerative medicine team, the efficacy of UC-MSC sheets for heart repair was further demonstrated in a porcine chronic myocardial ischemia model, in which the fibrosis area of the LV was clearly reduced, and cardiac function was remarkably promoted. In that study, the TdT-mediated dUTP nick-end labeling (TUNEL) and α-SMA immunostaining results showed a significant decrease in myocardial apoptosis and an increase in angiogenesis 28 days after UC-MSC sheets transplantation, and the HE and Masson staining revealed decreased fibrosis. Improved LVEF was also achieved and maintained for at least six months (unpublished data). Researchers also investigated the safety of UC-MSC sheets in rats, in which the oncogenicity, tumor promotion, sensitization, and tissue distributions after implantation were evaluated, as well as acute and long-term toxicity. This study proves that UC-MSC sheets are safe as a cell drug for ischemic myocardium treatment. The UC-MSC sheet makes it possible to improve the heart function of HF through medicine with universal quality instead of individualized medical technology.

### Clinical studies of cell sheets for cardiac tissue repair

Although direct injection of cells could mend small areas of damage, repairing the large areas of dead tissue resulting from a major coronary artery becoming locked requires lab-grown MSC or CM sheets. After preclinical studies using small and large animal models, the sufficient therapeutic efficacy of heart sheets should be provided through clinical studies and clinical trials before promoting their wide clinical applications. At present, clinical studies and trials based on SMs sheets and iPS-CM sheets are underway and can provide references for the evaluation of cell sheets constructed from other cell sources (Table 2).

**Table 2:** Clinical applications of SMs sheets and iPS-CMs sheets for the treatment of heart diseases

Cell type	Conditions	Study type	Numbers of patients	Follow-up time	Efficacy	Safety	Reference
SMs	An idiopathic DCM patient with an LVAD	First-in-human clinical trial	1	1 year	LVEF and LVDD were improved after 3 months	No life-threatening arrhythmia had occurred	[19]
					Wall motion improved first on the anterior and lateral surfaces and then on the other surface in the longer term		
					BNP levels declined and reached the normal range		
					The patient was able to discontinue using an LVAD and avoid cardiac transplantation		
	Cardiomyopathy patients without LVADs	Phase I clinical trial	15 ICM patients	More than 3 years	LVDD and LVEF showed significant improvement during 1 year	No lethal arrhythmias such as sustained VT and ventricular fibrillation were observed	[20]
					The NYHA classification had improved in all patients		
					End-systolic wall stress was significantly decreased at 6 months		
					Survival rate was 100% at 1 year and 90.9% at 3 years		
					No cardiac death event occurred within 3 years		
					Exercise capacity was improved via the 6MWD		
					A significant reduction of serum BNP level was observed		
					Reductions in PAP, PCWP, and PVR were observed		
					Reduction of LV wall stress was noted		
			12 DCM patients	More than 3 years	LVDD and LVEF were not statistically different after the treatment	2 patients with DCM developed congestive heart failure within 6 months	
					The survival rate was 90.0% at 1 year and 75.0% at 3 years		
					5 late cardiac deaths occurred within 3 years		
					Only limited efficacy was observed		
	Severe chronic HF due to ischemic heart disease	Phase II clinical study	7	26 weeks	LVEF was found improved or unchanged in 5 of the 7 patients	6 arrhythmia events and 3 SAEs occurred, but were all not drug-related	[15]
					The NYHA classification was found to improve in 6 of the 7 patients		
					A clear improvement in exercise tolerance was observed in most patients		
	End-stage ICM	Long-term clinical follow-up	23	Long-term	LVEF was found to improve or unchanged in 16 of the 23 patients at 6 months and the average increase was 4.9%	4 cases of cardiac unrelated mortality occurred	[62]
					The 1- and 5-year survival rates were 100% and 95% respectively		
					The 1- and 5-year freedom from composite events were 87% and 62% respectively		
					The NYHA classification was significantly improved		
					Substantial improvements in the serum BNP level and the 6MWD were found up to 3 years after the treatment		
					The hemodynamic variables did not significantly change for up to 3 years after the treatment		
	Pediatric DCM	Case study	1	6 months	LV volume remained unchanged	No arrhythmia or critical adverse events were observed	[16]
					LV contraction was sustainably ameliorated		
					Ross heart failure classification improved from the third to the first degree at 3 months, and no deterioration was observed		
					Cardiopulmonary exercise exhibited a trend of improvement via the 6MWD		
					Cold extremities, respiratory distress, and excessive sweating improved after sheet implantation		
iPS-CMs	ICM patients	A doctor-initiated clinical trial	3	6 months	The NYHA classification improved after 6 months: 2 patients from III to II and 1 patient from III to I	Three patients were all progressing well	–
					Three patients were all without disease progression during follow-up		

*SMs* skeletal myoblasts, *CM* dilated cardiomyopathy, *LVAD* left ventricular assist device, *LVEF* left ventricular ejection fraction, *LVDD* left ventricular end-diastolic dimension, *BNP* brain natriuretic peptide, *ICM* Ischemic cardiomyopathy, *NYHA* New York Heart Association, *6MWD* 6-min walk distance, *PAP* pulmonary artery pressure, *PCWP* pulmonary capillary wedge pressure, *PVR* pulmonary vascular resistance, *LV* left ventricle, *VT* ventricular tachycardia, *HF* heart failure, *SAEs* serious adverse events, *iPS-CMs* induced pluripotent stem cell-derived cardiomyocytes

#### Skeletal myoblasts (SMs) sheet

There have been many studies of SMs for heart repair since they were first proved useful for the treatment of ischemic cardiomyopathy in 1998 [56]. However, when SMs were delivered as injections, arrhythmia occurred occasionally, as the SMs were unable to form intercalated discs with adjacent cardiomyocytes [57–59]. Cell sheet technology provides an innovative formulation for the application of SMs in myocardial regeneration. Hata et al. demonstrated that autologous SM sheets attenuated cardiac remodeling and improved LV systolic and diastolic function in a pacing-induced canine heart failure model [60]. Miyagawa et al. demonstrated that implantation of SM sheets significantly induced angiogenesis and reduced fibrosis histologically, thereby preventing the deterioration of impaired myocardium in a porcine ischemic myocardium model [61]. Based on the positive effects in preclinical studies, autologous SM sheets were also transplanted to treat ICM, dilated cardiomyopathy (DCM) resulting in severe HF, as well as pediatric DCM in clinical studies and trials.

Sawa et al. conducted a first-in-man clinical trial using SM sheets to improve the cardiac function of a 56-year-old idiopathic DCM patient with a left ventricular assist device (LVAD) [19]. After the transplantation of cell sheets, his clinical condition improved markedly, leaving him without arrhythmia and able to discontinue the use of the LVAD and avoid cardiac transplantation. Because of the preclinical evidence and this positive clinical result, Sawa’s team started a phase I clinical trial at Osaka University Graduate School of Medicine, in which autologous SM sheets were transplanted to evaluate their safety and feasibility as the sole therapy for cardiomyopathy patients without LVADs [20]. As reported, 15 patients with ICM and 12 patients with DCM were enrolled for the implantation of myoblast sheets. During the follow-up period of more than six months, there were no procedure-related major complications, and most of the ICM patients showed remarkable symptomatic improvement in the New York Heart Association (NYHA) classification and exercise capacity improvement in the Six-Minute Walk Test. In the ICM patients, after implantation of cell sheets, reductions were noted in pulmonary artery pressure, pulmonary capillary wedge pressure, pulmonary vein resistance, and LV wall stress. For the DCM patients, only limited efficacy was observed. Based on the positive results of ICM treatment in the phase I clinical trial, Sawa’s team started an exploratory, prospective, multicenter, uncontrolled, open-label phase II study of autologous SM sheets for the treatment of severe chronic HF due to ischemic heart disease [15]. In this enrolled clinical trial with seven patients, no drug-related arrhythmia or other possible serious adverse events were observed, and almost all subjects showed symptomatic and exercise tolerance improvements. Moreover, the reported long-term outcome of autologous SM cell-sheet transplantation for 23 end-stage ischemic cardiomyopathy patients was a five-year survival rate higher than predicted [62]. Approximately 70% of the subjects presented improvement in LV ejection fraction six months after the treatment and achieved substantial LV unloading and improvements in LV systolic and hemodynamic functions. In addition to the adult heart disease treatment, there was also a clinical case report of the use of SM sheets in the treatment of pediatric DCM [16]. A three-year-old boy achieved recovery of cardiac function and improvements in exercise capacity after the implantation of SM sheets.

#### Induced pluripotent stem cell-derived cardiomyocytes (iPS-CMs) sheet

By the transduction of defined factors, human iPS cells were successfully established in 2007 [63]. iPS cells represented an unlimited source of CMs due to their differentiation potential, making them an ideal source for the preparation of cardiomyocytes and providing an alternative method of obtaining CM sheets. The safety and efficacy of iPS-CMs sheets for the treatment of heart diseases were investigated, both in animal models and in clinical trials [64–66]. Kawamura et al. stably cultured many highly pure human iPS-CMs in vitro and prepared cell sheets using these cells for preclinical study. The transplanted iPS-CMs sheets could remain on the heart’s surface for at least eight weeks and could significantly improve cardiac performance in a porcine ischemic cardiomyopathy model, mainly due to the paracrine effects of cytokines, the attenuation of LV remodeling, and the increase of neovascularization [67]. Moreover, when combining the cell sheets with the pedicled omental flap technique, the number of vessels and capillaries, the survival of transplanted iPS-CMs, and the amount of cytokine secreted can be further enhanced, indicating possibly better therapeutic effects [64, 68]. Sawa’s team from Osaka University carried out the first iPS cell-based heart cell transplantation in January 2020. Subsequently, a doctor-initiated clinical trial began, on the therapeutic effects of transplanting iPS-CM sheets in patients with ischemic cardiomyopathy (ICM). The clinical trial involves stringently evaluating risks, cancer probabilities, and the efficacy of transplanting 100 million cells per patient. Three patients (aged 52–77) had received transplantations of iPS-CM sheets by December 2020, with their ejection fraction around 30% at screening. Sawa reported that six months after transplantation, the three patients were progressing well without disease progression, and their NYHA heart function classifications had improved (3 → 2 or 3 → 1). iPS-CM can make meaningful connections with cardiomyocytes and provide paracrine factors that stimulate functional recovery of the myocardium. After transplantation, the cells on the degradable sheets attached to the surface of the patients’ hearts were expected to grow and secrete proteins that can regenerate blood vessels and improve cardiac function. We will continue to observe its long-term safety and effectiveness.

## Engineering technologies for cell sheets and 3D cell-dense tissue construction

### Cell sheet engineering

Cell sheet technology was developed by Okano’s team in 1990, using temperature-responsive culture dishes to harvest cells with sheet structure. Poly(N-isopropyl acrylamide), a temperature-responsive polymer with a lower critical solution temperature at 32 °C, is polymerized and grafted onto the culture dish surfaces by electron beam radical polymerization. Therefore, these intelligent surfaces can control the attachment and detachment of living cells with culture temperature changes from 37 to 32 °C. Cell sheet technology eliminates the necessity for proteolytic enzymes, which preserves the basal surface ECM proteins even after monolayer cell sheet detachment, and thus produces a basic building block that can be further manipulated to assemble more complex tissues and organs [69].

### 3D cell-dense tissue construction

Cell sheet technology provides a better formulation for cell therapy in heart disease curing, while cell-dense tissue based on cell sheets is an advanced cardiac tissue manufacturing strategy for heart tissue engineering. The 3D cardiac tissue can overcome the 2D cell sheets’ shortcomings of limited thickness and functionality and may be able to achieve heart tissue regeneration through tissue replacement. Due to the remaining ECM proteins, the cell sheets preserved the underlying adhesive properties, which was considered beneficial to transplantation into the target tissues [70]. This feature also facilitated the fabrication of 3D cardiac tissue by stacking cell sheets layer by layer [71]. The resulting construct resembled a dense cellular population with tight interlayer connections, including gap junctions, which facilitated the exchange of biomolecules and ions between layers and enabled an electrically synchronized contraction. In order to obtain a stable stacked cell sheet construct, a cell sheet manipulator was developed, to act as an ancillary tool for the temperature-responsive culture dishes [18, 71].

Although 2D cell sheets have been used successfully to construct 3D cardiac tissues, drawbacks remain with this technology. The most important issue to be addressed was the lack of vasculature, which hampered the diffusion of sufficient oxygen and nutrient into stacked cell sheets with high cell density, and thus limited the viable tissue thickness to three cellular layers [71–73]. The construction of functional vascularization within stacked cell sheets was found to be an efficient way to supply oxygen and nutrient to the engineered tissues and discard waste from them. Vascularization within stacked cell sheets can be improved by the inclusion of vascular cells or other cell types during fabrication [70, 72, 74]. These cells were incorporated in the form of either heterogeneous cell sheets or alternating mono-cultured homogeneous cell sheets. In this way, vascular cells were sandwiched between the cardiac cell sheet layers and promoted vascularization after transplantation. Engineered vascular networks are another powerful technique for vascularization; artificial interconnected microfluidic channels were introduced into the tissue by encapsulating and then melting away a sacrificial scaffold. After transplantation, in vivo vascularization can be achieved in these microchannel systems [75–77]. Besides these methods, in vitro artificial capillary beds provided another way to manufacture vascularized tissues [78]. In vitro artificial capillary beds can be generated by various advanced fabrication methods, such as micropatterning and in vitro perfusion, to control the spatial orientation of the vascular network and to enhance mass transport and perfusion.

In addition to vascularization, it is important to enhance the interlayer connectivity of the multilayered cell sheets, since these confluent cell layers are grown individually before assembly and likely have preferred cell connectivity within the same layer rather than between adjacent layers. Moreover, the electrophysiological connection between the 3D cardiac tissues and the host heart presents challenging research work for the future.

## Effectiveness and safety of MSCs sheets

### Mechanisms of action of MSCs sheets

Myocardial ischemia caused by various heart diseases can lead to a series of structural and functional changes in the heart [79, 80]. In the early stage, myocardial necrosis results in an influx of inflammatory cells, leading to the destruction of the collagen scaffolding, causing alteration of ventricular shape, regional thinning, and dilation of the ventricular wall in the infarcted areas. Over the following weeks to months, the viable myocardium is still challenged by the activation of proteases and elevated expression of cytokines, especially those that may induce cardiomyocyte apoptosis and increase the release of the proinflammatory factor. Thereafter, reactive myocyte hypertrophy, interstitial fibrosis, and left ventricular dilatation will cause adverse LV remodeling and finally lead to HF.

Many studies of MSCs suspension injections have revealed that they can take effect mainly through their paracrine effects, including the activation of endogenous precursors, promotion of neovascularization, favorable modulation of the extracellular matrix, and inhibition of apoptosis [54, 81–86]. The efficacy of MSC sheets is a combined effect of MSCs and the sheet structure. When transplanted onto the surface of the ischemic myocardium, MSC sheets can affect several stages of heart changes [55]. The sheet structure can prolong the retention and survival of MSCs, and thereby amplify their paracrine effects [40]. At the early stage, MSC sheets modulate inflammation to preserve the cardiomyocytes from acute injury. Furthermore, the abundant preserved ECM of MSC sheets prevents anoikis and apoptosis due to myocardial cell detachment from the ECM from occurring. Subsequently, the paracrine effects of MSCs bring about the release of various signals (e.g., cytokines, chemokines, growth factors, possibly exosomes or microparticles) into the surrounding tissue, which in turn promotes a series of restorative processes, including activation of endogenous precursors, neovascularization, inhibition of apoptosis, inhibition of hypertrophy, and favorable alterations of the ECM. Meanwhile, the ECM continuously produced from MSC sheets can serve as a bioactive scaffold for the host cells to graft and generate new epicardial tissue, providing mechanical support and routes for revascularization. The ECM scaffold was still maintained and worked even after the MSCs’ death. The reconstruction of myocardial tissue and the preserving ECM scaffold reduced LV wall stress, and thus prevented adverse LV remodeling, resulting in improved cardiac function [87].

### Safety and effectiveness concerns

Cell sheet structure enables cells to be concentrated at the transplantation site, with little metastasis to other organs, which helps to reduce some risks. The cell sources affect the therapeutic effect of the cell sheet. Usually, the cell sources are mainly autologous or allogeneic.

If autologous cells are used for cell sheet construction, there is no concern about immune rejection after transplantation. However, cell expansion and cell sheet preparation usually take one month or more, which is unsuitable for patients in urgent need of cell sheet transplantation. Many other risks should be taken into consideration. Firstly, the patient, who is also the donor of cells, needs strict virus control. Secondly, as an in vitro culture process may introduce pathogens and sensitizers, strict process control and system verification are necessary. Moreover, an autologous cell sheet is a medical technology rather than a medical product and is affected by individual differences, including age and medical history, which would affect safety and efficacy. Therefore, there is a requirement for sufficient individual studies to be undertaken, to accumulate sufficient data with which to amend the deflection caused by individual differences.

UC-MSCs as allogeneic cells have attracted much attention because of their rapid self-renewal, multipotent, painless collection, and compliance with standard amplification. Allogeneic cells combined with cell cryopreservation technology is more conducive to the production of large-scale cell sheets, which can partly eliminate the differences between individuals from cell source. Meanwhile, the tumorigenicity, shelf stability, and other quality data can be confirmed in advance. However, although MSCs have low immunogenicity, they are not completely free from the risk of immune rejection, especially in the acute phase. Patients may require anti-immune drugs in the acute stage after cell sheet transplantation. In addition, donor selection and informed consent procedures must follow the rules.

MSC sheets are mainly helpful for heart repair, while SM sheets and iPS-CM sheets exhibit greater potential for functional replacement of the myocardium. However, the risk of causing post-transplant arrhythmia in patients and the tumorigenicity of iPSs are still of concern. Long-term observation is needed, to evaluate safety and effectiveness.

Scaffold-based cardiac tissues are also widely used in heart repair [72]. Compared with them, cell sheets contain no exogenous or synthetic materials, so concerns about the potential immunogenicity of the scaffold material are abolished [40, 88]. Moreover, the remaining ECM proteins of cell sheets retain adhesive properties, avoiding the need for sutures or the usage of adhesives during transplantation [88]. Engineering tissues constructed by cell sheets are denser and possess cell-to-cell connections and gap junctions, which can create a cell growth environment more like the original myocardial tissue, and thus achieve more effective heart repair [87, 88].

## Discussion

Cardiac regeneration is a rapidly evolving field of research. Among them, cell sheets constructed with tissue engineering have been paid more and more attention in the field of tissue and organ repair, as they can build two-dimensional and three-dimensional organizations. Cell sheets with two-dimensional structures improve cell retention and utilization compared with cell suspension injection in cell therapy. After more than 30 years of development, cell sheet technology has made significant breakthroughs and progress both in fundamental research and clinical applications. So far, there have been more than 70 cases of cell sheets clinical practice in treating heart disease. According to the results of previous research, the following points should be the main focus of attention: suitable cell sources and cell type, preparation of cell sheet with stable quality, the indication of cell sheet treatment, evaluation of long-term safety and effectiveness after cell sheet transplantation, construction of three-dimensional tissues, etc.

For the cell source, adult stem cell transplantation’s contribution to improving cardiac function is due to its paracrine effects. Autologous stem cells can avoid immune rejection after transplantation. However, the long preparation waiting time, non-standardized preparation and quality control process, and uncontrollable individual differences make autologous cell sheets that only can be used as a medical technology rather than a medical product. Allogeneic cells combined with cell cryopreservation technology are more suitable to prepare large-scale cell sheets as off-the-shelf products. Nevertheless, strict donor selection and the potential risk of immune rejection should be taken seriously. Among various cell types, UC-MSCs, as allogeneic cells have attracted much attention. UCMSCs have the characteristics of stem cells like MSCs derived from bone marrow, adipose tissue, and other tissues. Moreover, UCMSCs can be frozen for long-term storage, and cell banks can be established and managed with a unified quality standard, to provide safe and high-quality cells for cell sheet preparation [89]. Whereas allogeneic iPS-CMs are also a potential cell source for cell sheet manufacture. As iPS-CMs possess not only the paracrine effects but also the function of cardiomyocytes, thus exhibit greater potential for functional replacement of the myocardium. A clinical trial of the iPS-CM sheet transplantation confirmed the efficacy and safety of ICM patients at Osaka University in the period from 2019 to 2023.

The applications of cell sheets in the treatment of heart disease were now focused on the ICM caused by the narrowing of the coronary arteries and DCM caused by the damage of the myocardium. From the reported clinical data, it can be found that a significant difference exists in the efficacy of the treatment of ICM and DCM patients. For ICM patients, the effective rate was nearly 70%, in which LVEF, NYHA classification, and other cardiac function indexes were significantly improved after cell sheet transplantation. However, there was no significant efficacy for DCM patients, in which only a small number of patients benefited from cell sheet transplantation. These clinical results suggest that cell sheets may be more effective for patients with poor peripheral circulation. With further studies of the efficacious mechanism and the increase in clinical cases, more information for the precise selection of indications will be obtained.

The safety after cell sheet transplantation is another concern. Arrhythmia is a common adverse reaction to the previous injection of cell suspension. When the cells are transplanted to the heart surface as cell sheets, arrhythmia seems to be avoided. However, the safety of cell sheets is concluded only by the limited clinical data of about 70 cases. Arrhythmia is the most serious risk factor for the clinical application of cell sheets, and it needs continuous attention during clinical trials and clinical applications.

Although transplantation of a monolayer cell sheet could successfully mend ischemic areas of damaged myocardial tissue, repairing the large areas of dead tissue will require lab-grow multi-layer cell sheets. It is also a great challenge to construct multi-layer and functional tissues because most bioreactors cannot supply enough nutrients and oxygen to the growing thickness of tissue. Usually, growth in a bioreactor typically stops once the tissue is about 3–5 cell layers thick. Beyond this thickness, the innermost cells are far from the supply of fresh growth medium. Approaches to solving the problem include co-culturing endothelial cells with other cell types during cell sheet construction, sandwiching endothelial cells between cell sheet layers [18], and introducing artificial channels into tissue via a sacrificial scaffold, or in vitro artificial capillary beds [90] to create a three-dimensional tissue. Meanwhile, the three-dimensional tissues need to keep oxygenated during implantation and for some weeks afterward, until the patient’s won vessels begin to colonize the implant.

## Conclusion

Cell sheet technologies are developing rapidly, and numerous preclinical and clinical studies are underway. Cell sheets can be constructed by different cell types from various sources, and further manipulated to prepare a 3D cell-dense tissue. The safety and efficacy of cell sheets for heart repair have been well demonstrated in numerous animal experiments and tested in several clinical studies. However, challenges still exist. In future studies, special attention should be focused on the following sections: selection of suitable cell sources and types for better safety and efficacy, stable preparation and quality control of cell sheets for quantity production, construction of a 3D cell-dense tissue with functional vessels for tissue replacement, a precise selection of clinical indications for better efficacy, and long-term follow-up of more cases after cell sheet transplantation for better assessment of the safety.

## Data Availability

Not applicable.

## Abbreviations

HF: Heart failure
MI: Myocardial ischemia
LV: Left ventricle
2D: Two-dimensional
ECM: Extracellular matrix
MSCs: Mesenchymal stem cells
iPS-CMs: Induced pluripotent stem cell-derived cardiomyocytes
3D: Three-dimensional
CMs: Cardiomyocytes
BM-MSCs: Bone marrow-derived mesenchymal stem cells
ICM: Ischemic cardiomyopathy
AD-MSCs: Adipose-derived mesenchymal stem cells
LVEF: Left ventricular ejection fraction
UC-MSCs: Umbilical cord-derived mesenchymal stem cells
ICR: Institute of Cancer Research
HNA: Human nuclei antigen
TUNEL: TdT-mediated dUTP nick-end labelling
SMs: Skeletal myoblasts
DCM: Dilated cardiomyopathy
LVAD: Left ventricular assist device
NYHA: New York Heart Association
LVDD: Left ventricular end-diastolic dimension
BNP: Brain natriuretic peptide
6MWD: 6-Minute walk distance
PAP: Pulmonary artery pressure
PCWP: Pulmonary capillary wedge pressure
PVR: Pulmonary vascular resistance
VT: Ventricular tachycardia
SAEs: Serious adverse events
